# Identification of the Intragenomic Promoter Controlling Hepatitis E Virus Subgenomic RNA Transcription

**DOI:** 10.1128/mBio.00769-18

**Published:** 2018-05-08

**Authors:** Qiang Ding, Ila Nimgaonkar, Nicholas F. Archer, Yaron Bram, Brigitte Heller, Robert E. Schwartz, Alexander Ploss

**Affiliations:** a Department of Molecular Biology, Lewis Thomas Laboratory, Princeton University, Princeton, New Jersey, USA; b Division of Gastroenterology & Hepatology, Department of Medicine, Weill Medical College of Cornell University, New York, New York, USA; Virginia Polytechnic Institute and State University

**Keywords:** hepatitis E, hepatitis E virus, viral hepatitis, viral replication

## Abstract

Approximately 20 million hepatitis E virus (HEV) infections occur annually in both developing and industrialized countries. Most infections are self-limiting, but they can lead to chronic infections and cirrhosis in immunocompromised patients, and death in pregnant women. The mechanisms of HEV replication remain incompletely understood due to scarcity of adequate experimental platforms. HEV undergoes asymmetric genome replication, but it produces an additional subgenomic (SG) RNA encoding the viral capsid and a viroporin in partially overlapping open reading frames. Using a novel transcomplementation system, we mapped the intragenomic subgenomic promoter regulating SG RNA synthesis. This *cis*-acting element is highly conserved across all eight HEV genotypes, and when the element is mutated, it abrogates particle assembly and release. Our work defines previously unappreciated viral regulatory elements and provides the first in-depth view of the intracellular genome dynamics of this emerging human pathogen.

## IMPORTANCE

HEV is an emerging pathogen causing severe liver disease. The genetic information of HEV is encoded in RNA. The genomic RNA is initially copied into a complementary, antigenomic RNA that is a template for synthesis of more genomic RNA and for so-called subgenomic RNA. In this study, we identified the precise region within the HEV genome at which the synthesis of the subgenomic RNA is initiated. The nucleotides within this region are conserved across genetically distinct variants of HEV, highlighting the general importance of this segment for the virus. To identify this regulatory element, we developed a new experimental system that is a powerful tool with broad utility to mechanistically dissect many other poorly understood functional elements of HEV.

## INTRODUCTION

In 1983, the first experimental evidence was provided for an additional waterborne hepatitis agent distinct from hepatitis A virus (HAV) that was eventually named hepatitis E virus (HEV) (1). HEV is recognized as a leading cause of fecally-orally transmitted viral hepatitis globally, causing an estimated 60,000 deaths and 3,000 stillbirths a year (2). HEV prevalence is increasing in both industrialized and developing nations and causes a suite of problems, including chronic infection in immunocompromised patients and up to 30% mortality in pregnant women in the third trimester. The HEV genome was first cloned in 1990 (3), enabling inferences of the genome organization and putative functions of virally encoded gene products based on homology to related viruses (4). Since then, significant progress has been made in our understanding of the molecular virology of this virus; however, our knowledge still contains numerous gaps that are hampering the development of direct-acting antiviral therapeutics (4).

HEV is a positive-sense (+), single-stranded, quasienveloped icosahedral RNA virus belonging to the *Hepeviridae* family (2, 5). The 7.2-kb genome has a 7-methylguanosine cap structure in the 5′ untranslated region (5′ UTR) and is polyadenylated at the 3′ end. It is generally accepted that HEV contains three open reading frames (ORFs), although a recent study hinted that endoplasmic reticulum stress promotes cap-independent, internal initiation-mediated translation of an additional HEV protein, ORF4, in one genotype of the virus (6). ORF1 codes for a nonstructural polyprotein comprised of a methyltransferase, putative papain-like cysteine protease, X domain (characterized as a macro domain [7–9]), RNA helicase, and RNA-dependent RNA polymerase (RdRp) (2). ORF2 is the viral capsid protein and is involved in virion assembly, interaction with the host cell, and immunogenicity. Finally, the proline-rich ORF3 is the smallest ORF of the HEV genome, sharing most of its bases with ORF2. We have recently provided evidence that ORF3 is a viroporin, or virally encoded ion channel, that is essential for release of infectious particles from infected cells (10).

Following viral entry into host cells and uncoating of the viral genome, the (+) RNA is translated by host ribosomes into a polyprotein containing the nonstructural enzymes of the virus, including the RdRp. This HEV RdRp then transcribes the (+) RNA into a complementary negative-sense (−) RNA. Consistent with presumed asymmetric genome replication, the full-length (FL) (−) strand genome serves as a template for more (+) RNA and for synthesis of the SG RNA(s) encoding ORFs 2 and 3. Transcription of the (+) and (−) RNA is initiated at secondary RNA structures at the respective 5′ and 3′ UTRs. Nucleotides on the stem-loop RNA structure in the junction region (JR) between the stop codon of ORF1 and the start codons of ORFs 2 and 3 have been implicated in viral genome replication (11). However, due to the lack of adequate experimental systems, it remains unclear where precisely the *cis*-acting element regulating subgenomic (SG) RNA synthesis is and what the kinetics and dynamics of the different viral RNAs [FL (+), (−) strand and SG] are. These are critical questions, and answering them will provide important insights into the mechanism of HEV genome replication.

In this study, we aimed to identify the *cis*-acting element functioning as the promoter to direct HEV subgenomic RNA synthesis. We devised a novel experimental system enabling us to interrogate the functional relevance of specific nucleotides within the FL genome by complementing the ORF1 function in *trans*. Using this novel transcomplementation system, we precisely mapped the region within the viral (−) RNA that is critical for transcription of the HEV subgenome. This subgenomic promoter (SgP) spans a 44-nucleotide region from the 3′ end of the ORF1 to the transcriptional start site (TSS) of ORF3 (11, 12) and a 9-nucleotide genetic element from the TSS to the translational start site of ORF3, thus extending beyond the junction region which has been previously implicated in the transcriptional control of the HEV subgenomic RNA (13). The sequence of this SgP is highly conserved across the eight, genetically diverse genotypes of the *Orthohepevirus A* species. Introducing mutations within the SgP in the HEV genome abrogated the transcription of the RNA encoding ORF2 and ORF3 and thus severely impaired assembly and release of infectious virions. Our work not only defines precisely the *cis*-acting elements controlling critical parts of the HEV replicative cycle but also adds to a more comprehensive view of the mechanism of HEV RNA replication.

## RESULTS

### Development of a transcomplementation system to analyze functionally *cis*-acting elements critical for HEV genome replication.

**FIG 1:**
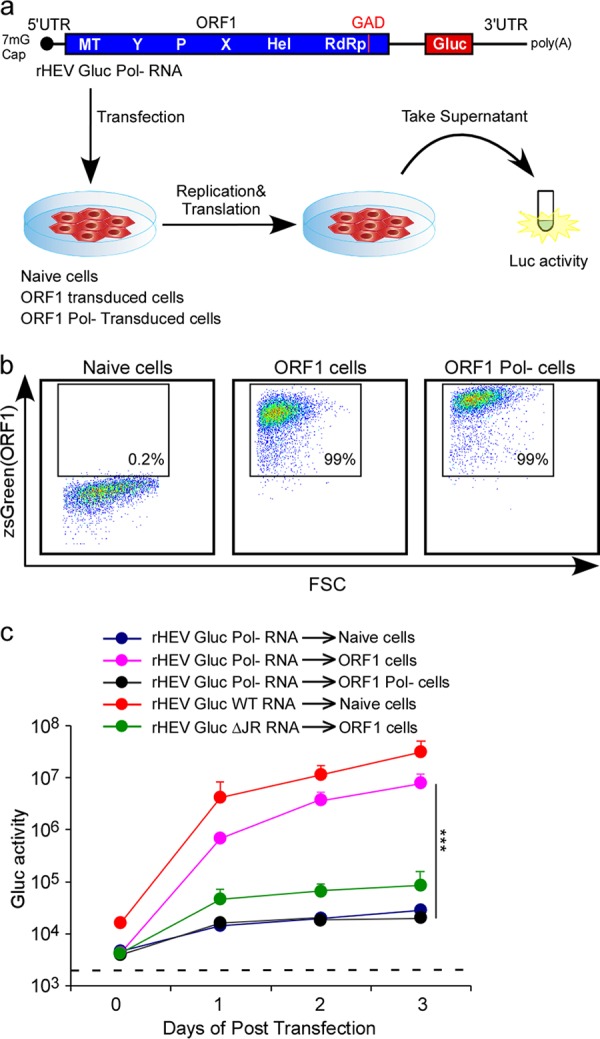
HEV ORF1 is able to function in *trans* to replicate HEV RNA. (a) Schematic representation of the ORF1 transcomplementation system. (b) Representative flow cytometry plots demonstrating efficient ORF1 expression. HepG2C3A cells were transduced with pLVX-ORF1-IRES-zsGreen (wild type [wt] or GAD mutant) or not transduced. Flow cytometric analysis was performed 3 days following transduction to quantify the frequencies of ORF1-expressing cells. FSC, forward scatter. (c) Replication kinetics of HEV RNA in ORF1 transcomplemented HepG2C3A cells. Cell culture supernatants from naive HepG2C3A cells, or HepG2C3A cells transduced with HEV ORF1 or its GAD mutant, were collected at the indicated time points posttransfection with rHEVΔORF2/3[Gluc] Pol- RNA or RNA from its mutants, and *Gaussia* luciferase (Gluc) activity was quantified. Values are means plus standard deviations (SD) (error bars) (*n* = 3). Values that are significantly different (*P* < 0.001 by two-tailed Student’s *t* test) are indicated by the bar and three asterisks.

To map the intragenomic subgenomic promoter (SgP) that is critical for SG RNA synthesis off the FL (−) strand, we devised an experimental system in which HEV RNA transcription is uncoupled from protein translation. Following our previously established successful transcomplementation approach for studying HEV ORF3’s viroporin function (10), we expressed lentivirally a wild-type (wt) or polymerase-defective (GAD or Pol−) version of ORF1 in HepG2C3A hepatoma cells (Fig. 1a and b). Flow cytometric quantitation of the bicistronically expressed zsGreen reporter confirmed that both constructs were expressed equally well in HepG2C3A cells (Fig. 1b). wt or Pol− ORF1-expressing cells were subsequently transfected with *in vitro*-transcribed RNA from a recombinant, Pol− version of an HEV genome derived from the Kernow C1/p6 genome (14) in which ORF2 and ORF3 are replaced by a secreted version of *Gaussia* luciferase (Gluc) (14), termed rHEVΔORF2/3[Gluc] Pol- (Fig. 1a). Gluc activity as a measure for the efficiency for RdRp-mediated transcription initiated at the elusive intragenomic SgP was quantified in the culture supernatants on days 1, 2, and 3 after RNA transfection. Transfection of the wt rHEVΔORF2/3[Gluc] into naive HepG2C3A cells led to a ca. 1,000-fold increase in luminescence, whereas transfection of the Pol− genome expectedly did not augment Gluc activity (Fig. 1c). Importantly, transfection of rHEVΔORF2/3[Gluc]-Pol- into wt but not Pol− ORF1-expressing cells rescued Gluc expression, demonstrating that HEV RNA polymerase-dependent replication can be rescued in *trans*. These differences are not due to differences in the expression between the wt and Pol− ORF1. In fact, Western blotting analysis of epitope-tagged ORF1 demonstrated that the wt and Pol− mutant proteins are expressed at equivalent levels (see Fig. S2 in the supplemental material). Of note, insertion of the Flag tag at the C terminus of ORF1 did not impair its ability to rescue replication of rHEVΔORF2/3[Gluc]-Pol- RNA in *trans* (Fig. S2). Consistent with previous reports (13), deletion of the JR almost completely abrogated Gluc activity, presumably due to the inability of the HEV RdRp to produce an SG RNA encoding the reporter gene.

### Fine-mapping of the intragenomic region upstream of the TSS controlling synthesis of the HEV subgenomic RNA.

**FIG 2:**
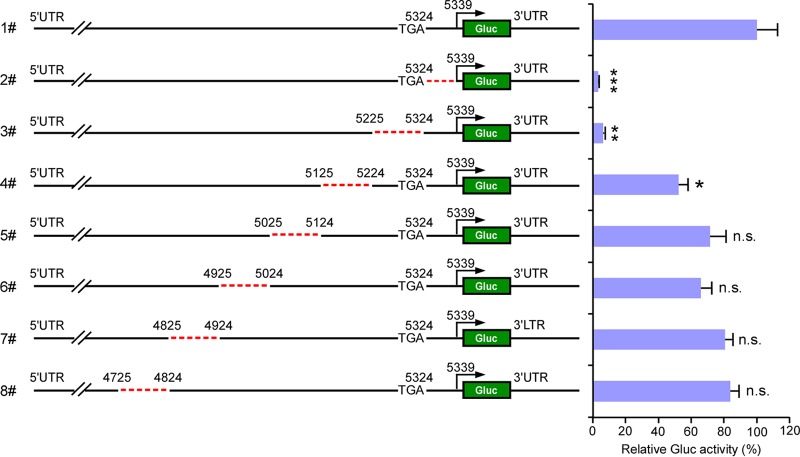
Mapping the putative promoter region required for subgenomic RNA synthesis. A series of truncated rHEVΔORF2/3[Gluc] Pol- mutant constructs were generated, and the *in vitro*-transcribed RNA was transfected into HepG2C3A cells expressing ORF1. Two days posttransfection, supernatants were collected, and *Gaussia* luciferase activity was quantified. The data are presented as the percentage of *Gaussia* luciferase activity relative to that of the full-length rHEVΔORF2/3[Gluc] Pol-. The numbering denotes the positions of the Kernow C1/p6 viral genome. Values are means plus SD (*n* = 3). Values that are significantly different by one-way ANOVA are indicated by asterisks as follows: *, *P* < 0.05; **, *P* < 0.01; ***, *P* < 0.001. Values that are not significantly different (n.s.) by one-way ANOVA are also indicated.

Uncoupling of HEV RNA transcription from protein translation opened opportunities to map the SgP producing the SG RNA encoding HEV ORF2 and ORF3. Sequences identical or very similar to those at the respective 5′ and 3′ UTRs forming secondary RNA structures that regulate transcription of the (+) and (−) RNA are not present in other parts of the genome. Thus, we followed an unbiased approach and first screened a larger region upstream of the subgenomic RNA transcriptional start site (11, 12) for the presence of functionally relevant elements. First, we generated rHEVΔORF2/3[Gluc]-Pol- harboring approximate 100-nucleotide (nt) deletions between positions 4725 nt to 5324 nt (ORF1 stop codon) (Fig. 2). These mutant RNAs were subsequently transfected into wild-type ORF1-expressing HepG2C3A cells, and reporter activity in the supernatant was monitored. While most deletions did not affect or only minimally affected Gluc activity, deleting residues 5225 to 5324 nt (row 3) reduced the activity of the luminescent reporter to levels similar to those of the ΔJR mutants (row 2). These observations indicated that the subgenomic promoter was likely contained in this region.

**FIG 3:**
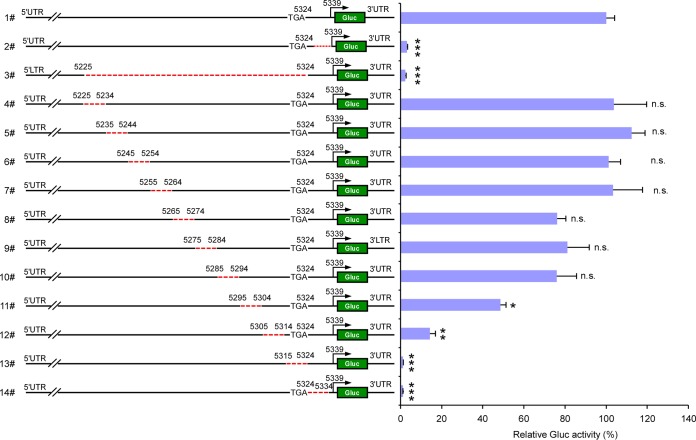
Identification of the minimal putative promoter region upstream of the TSS critical for subgenomic RNA synthesis. The truncated mutant viral RNA was transfected into HepG2C3A cells expressing ORF1. Two days posttransfection, supernatants were collected, and *Gaussia* luciferase activity was quantified. The data are presented as the percentage of *Gaussia* luciferase activity relative to that of the full-length rHEVΔORF2/3[Gluc] Pol-. The numbering denotes the positions of the Kernow C1/p6 viral genome. Values are means plus SD (*n* = 3). *, *P* < 0.05; **, *P* < 0.01; ***, *P* < 0.001; n.s., not significantly different by one-way ANOVA.

To more accurately map the relevant sequence, we created additional mutant rHEVΔORF2/3[Gluc]-Pol- genomes lacking 10-nt segments between positions 5225 to 5339 nt (Fig. 3). Similarly, these genomes were transfected into HepG2C3A cells lentivirally transduced with native ORF1. Whereas the deletions within the region from positions 5225 to 5294 resulted in levels of secreted Gluc comparable to those of the unmodified rHEVΔORF2/3[Gluc]-Pol- genome, the reporter activity was decreased by 50% when nucleotides 5295 to 5304 were deleted. Of note, deletion of nt 5305 to 5314 decreased Gluc activity by 85%, and deletions of nt 5315 to 5324 and nt 5324 to 5334 (containing part of the JR segment) decreased activity by >95%. Collectively, these data suggest that nucleotides 5295 to 5338 constitute a critical region of the subgenomic promoter.

### Deletion of the subgenomic promoter upstream of TSS does not affect the abundance of the HEV negative strand.

**FIG 4:**
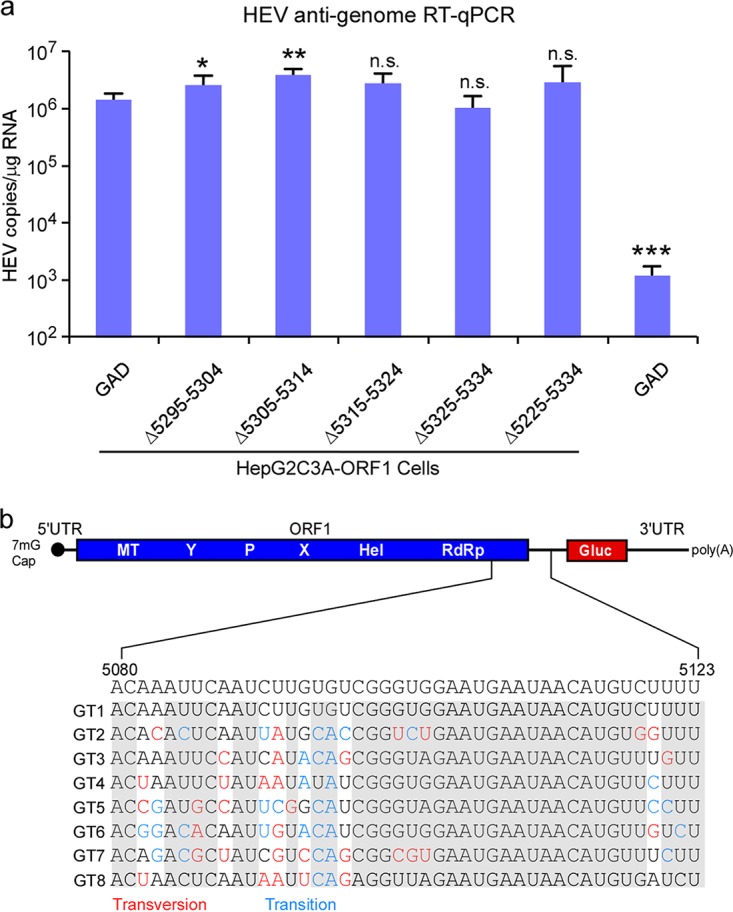
Synthesis of antigenomic HEV RNA is not affected by deletions in the putative promoter region. (a) HEV ORF1 transduced HepG2C3A cells were transfected with the indicated truncated rHEVΔORF2/3[Gluc] Pol- RNA genome. After 2 days, cells were washed, and intracellular total RNA was extracted and subjected to HEV negative-strand RNA-specific RT-qPCR assay to measure the abundance of antigenome. The numbering denotes the positions of the Kernow C1/p6 viral genome. Values are means plus SD (*n* = 3). *, *P* < 0.05; **, *P* < 0.01; ***, *P* < 0.001. n.s., not significantly different by one-way ANOVA. (b) Consensus sequences of the putative promoter across genotypes 1 to 8. M73218 (genotype 1a, Burma strain) was used as the reference strain for numbering. The consensus sequences (identity of >60%) are shaded. The red or blue colors denote the transversion or transition from the reference sequence.

Conceivably, deleting these—albeit—small regions within the HEV genome could possibly affect the efficiency of HEV (−) strand RNA synthesis and/or stability of the negative strand, thereby indirectly affecting SG RNA synthesis. To rule out this possibility, we devised a sensitive negative-strand RNA quantitative reverse transcription-PCR (RT-PCR) detection assay. Using this strand-specific quantitative RT-PCR assay (Fig. S1), we demonstrate that (−) HEV RNA was equivalent for all mutant genomes transfected into HepG2C3A expressing wt ORF1 (Fig. 4a). By comparison, HepG2C3A transfected with a Pol− genome harbored about 1,000-fold fewer HEV (−) strand RNA copies. Thus, the observed reductions in the reporter activity in the rHEVΔORF2/3[Gluc]-Pol- genome harboring 10 nucleotide deletions between nucleotide 5295 to 5334 cannot simply be attributed to differences in the amount of the (−) strand RNA template. Rather, these data provide further evidence that these deletions functionally inactivate a *cis*-acting element within the viral genome critical for regulating transcription of subgenomic HEV RNA.

10.1128/mBio.00769-18.1 FIG S1  Strand-specific RT-qPCR assay to quantify the HEV negative-strand RNA genome. (a) Schematic illustration of the positions of the primers in the RT-qPCR assay. For the RT primer (PU-O-4508 [see Table 2]), a specific tag sequence was added at the 5′ end for RT to generate cDNA derived from negative viral RNA genome. For PCR primers, the forward (Fwd.) primer (PU-O-4509) and reverse (Rev.) primer (PU-O-4125) were used in this study for qPCR assay. (b) The standard curves were generated by quantifying *in vitro*-transcribed RNA using the strand-specific RT-qPCR. cDNAs synthesized by reverse transcription of negative-strand viral RNA genome in the presence or absence of a fixed amount of *in vitro* transcript positive- or negative-strand viral RNA genome using tagged RT primer were quantified using qPCR. The experiment was performed in triplicate, and mean threshold cycle (*C*_*T*_) values with standard deviation were plotted against RNA absolute copy number. The correlation coefficients of the qPCRs are presented below each curve. Download FIG S1, TIF file, 3.3 MB. Copyright © 2018 Ding et al. 2018 Ding et al. This content is distributed under the terms of the Creative Commons Attribution 4.0 International license.

10.1128/mBio.00769-18.2 FIG S2  HEV Flag-tagged ORF1 is able to function in *trans* to replicate HEV RNA. (a) Schematic representation of the ORF1 transcomplementation system. (b) Representative flow cytometry plots demonstrating efficient ORF1Flag transduction. HepG2C3A cells were transduced with pLVX-ORF1Flag-IRES-zsGreen (WT or GAD mutant) or not transduced. Flow cytometric analysis was performed 3 days following transduction to quantify the frequencies of ORF1-expressing cells. (c) The ORF1 Flag (WT or GAD mutant)-transduced HepG2C3A cells were subjected to immunoblotting to analyze the expression of ORF1Flag with Flag antibody. (d) Replication kinetics of HEV RNA in ORF1 transcomplemented HepG2C3A cells. Cell culture supernatants from naive HepG2C3A cells or HepG2C3A cells transduced with epitope-tagged HEV ORF1 or its GAD mutant were collected at the indicated time points posttransfection with rHEVΔORF2/3[Gluc] Pol- RNA or its mutants RNA, and *Gaussia* luciferase activity were quantified. Data represent the means plus SD (*n* = 3). ***, *P* < 0.001 by two-tailed Student’s *t* test. Download FIG S2, TIF file, 5.6 MB. Copyright © 2018 Ding et al. 2018 Ding et al. This content is distributed under the terms of the Creative Commons Attribution 4.0 International license.

### The intragenomic subgenomic promoter is highly conserved and controls synthesis of HEV subgenomic RNA,

**FIG 5:**
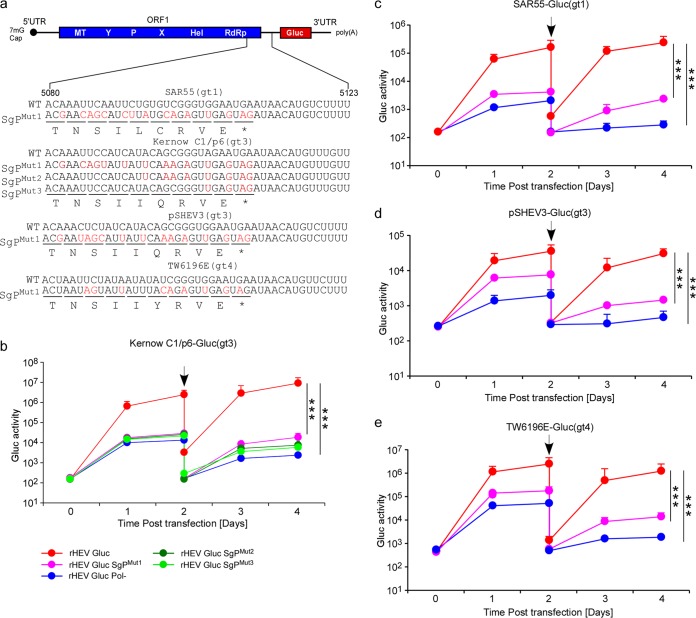
Mutations in the intragenomic promoter significantly disrupt HEV subgenomic RNA transcription. (a) Schematic diagrams of the putative promoter (WT) and the SgP mutants (SgP^mut1^, SgP^mut2^, and SgP^mut3^). As for the promoter impaired mutant, synonymous mutations are introduced in the ORF1 coding region. M73218 (genotype 1a, Burma strain) was used as the reference strain for numbering. (b to e) rHEVΔORF2/3[Gluc] WT, SgP mutant(s), or GAD mutant RNA were delivered into HepG2C3A cells. Cell culture medium was collected at the indicated time points, and *Gaussia* luciferase activity of Kernow C1/p6-Gluc (b), SAR55-Gluc (c), pSHEV3-Gluc (d), and TW6196E-Gluc (e) was determined. Black arrows indicate the time point when cells were washed at day 2 posttransfection, and the *Gaussia* luciferase before and after wash were plotted. Values are means plus SD (*n* = 3). ***, *P* < 0.001 by two-tailed Student’s *t* test.

Sequence alignments of the SgP region (5295 to 5338 nt of the Kernow C1/p6 viral genome, corresponding to nucleotides 5080 to 5123 nt M73218 [genotype 1a, Burma strain] reference genome) showed that this *cis*-acting element is highly conserved across genetically diverse HEV genotypes 1 to 8 (Fig. 4b), which is consistent with the notion of the critical significance of this segment for an essential step in the HEV replication cycle. Next, we aimed to validate the importance of SgP for the HEV life cycle in the context of a full-length infectious viral genome. In the first step, we introduced synonymous mutations in 14 nucleotides in the C-terminal end of the ORF1 coding sequence of the Kernow C1ΔORF2/3[Gluc] which overlaps the newly mapped SgP (Fig. 5a). Consistent with our previous data, Gluc activity was reduced 100- to 500-fold in supernatants of cells transfected with the resultant Kernow C1ΔORF2/3[Gluc] SgP^mut1^ (magenta line) compared to the parental sequence (Fig. 5b). To determine which of the nucleotides within this region are particularly important, we created two additional mutant genomes in introduced synonymous mutations in only 8 (rHEV Gluc sgP^Mut2^) or 4 nucleotides (rHEV Gluc SgP^Mut3^). Similar to the rHEV Gluc SgP^Mut1^ harboring 14 synonymous mutations, Gluc activity was reduced 500- to 1,000-fold for both the sgP^Mut2^ (Fig. 5b, dark green line) and sgP^Mut3^ genomes (Fig. 5B, light green line), suggesting that even minor changes in this region affect transcription of the SG RNA.

To provide direct experimental evidence that mutations in the SgP also affect SG RNA transcription of other, genetically diverse HEV genotypes, we expanded our analysis to SAR55 (genotype [gt] 1) (12, 15), pSHEV3 (gt 3) (16), and TW6196E (gt 4) (17). To enable the same simple readout, we constructed a panel of HEV reporter genomes for these genotypes using the same rHEVΔORF2/3[Gluc] configuration (Fig. S3a to d). Transfection of *in vitro*-transcribed RNA into Huh7.5 hepatoma cells demonstrated that all reporter genomes are replication competent (Fig. S3). Of note, insertion of the S17 sequence that was shown to boost replication of the Kernow C1 p6 genome (14) (Fig. S3) also augmented replication of other HEV genotypes. Making use of this panel of genetically diverse viral reporters, we introduced synonymous mutations in the ORF1 coding sequences of subgenomic promoter region (Fig. 5a). Similar to the Kernow C1ΔORF2/3[Gluc], SgP^Mut1^ luciferase activity was also 100- to 300-fold decreased for the SAR55 (Fig. 5c), pSHEV3 (Fig. 5d), and TW6196E (Fig. 5e) ΔORF2/3[Gluc] SgP^Mut1^ compared their respective wt parental sequences. Expectedly, introducing mutations in the catalytically active site of the RdRp (Pol−) reduced luciferase activity to background levels.

10.1128/mBio.00769-18.3 FIG S3  Generation and characterization of HEV reporter genomes. Schematic diagrams of the rHEVΔORF2/3[Gluc] genomes. S17 sequences were inserted into the X domain of ORF1 as described. GAD mutant was generated as the negative control. rHEVΔORF2/3[Gluc] WT, S17 inserted or GAD mutant RNA were delivered into Huh 7.5 cells. Cell culture medium was collected after 4 days transfection, and *Gaussia* luciferase activity was determined. Data represent the means plus SD. (*n* = 3). *, *P* < 0.05; ***, *P* < 0.001 by two-tail Student’s *t* test. Download FIG S3, TIF file, 10.5 MB. Copyright © 2018 Ding et al. 2018 Ding et al. This content is distributed under the terms of the Creative Commons Attribution 4.0 International license.

**FIG 6:**
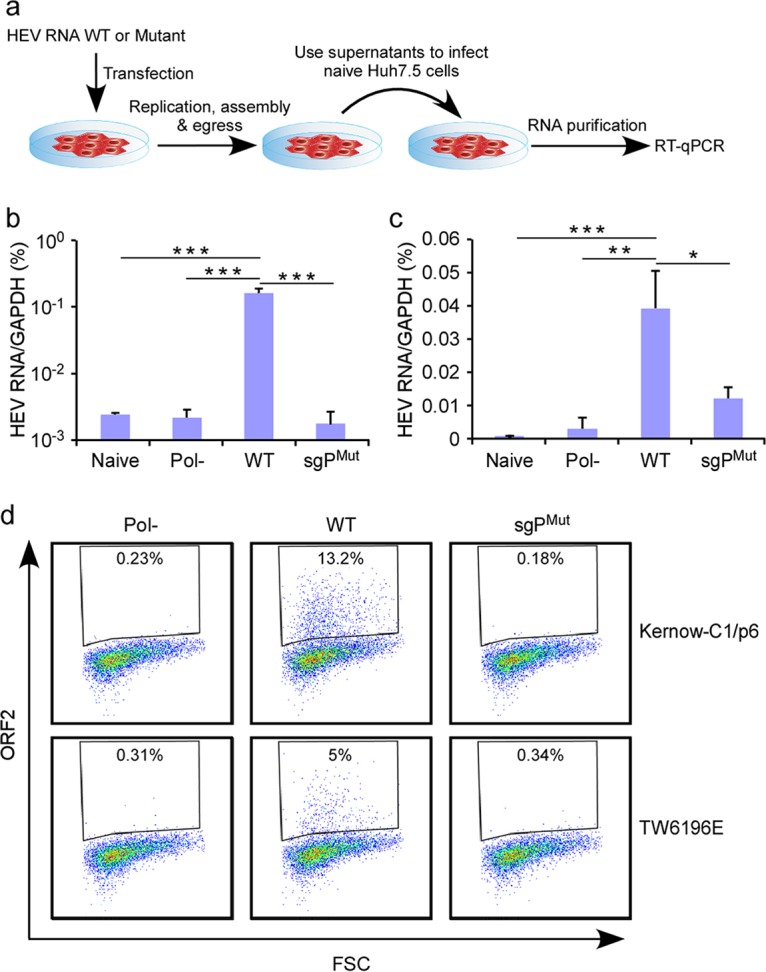
Full-length genome mutated in the intragenomic promoter is significantly impaired in its ability to produce the infectious virus. (a) Schematic diagrams of the putative promoter (WT) and the SgP mutant (SgPmut). As for the promoter impaired mutant, synonymous mutations are introduced in the ORF1 coding region. (b and c) Transfection of *in vitro*-transcribed WT, SgPmut or GAD (Pol-) RNA of Kernow-C1/p6 (gt 3), TW6196E (gt 4) into Huh7.5 cells. Cell lysate supernatant was collected after 5 days transfection to infect naive Huh7.5 cells. Quantification of HEV RNA Kernow-C1/p6 (b) and TW6196E (c) 3 days following infection by quantitative RT-PCR. Values are means plus SD (*n* = 4). *, *P* < 0.05; **, *P* < 0.01; ***, *P* < 0.001 by one-way ANOVA. GAPDH, glyceraldehyde-3-phosphate dehydrogenase. (d) Representative flow cytometric plots of Huh7.5 cells infected with Kernow-C1/p6 (top) or TW6196E (bottom) 5 days following infection.

If HEV SG RNA synthesis were truly dependent on this *cis*-acting element, then mutations in this region abrogating this function would severely impair particle production and release, as the SG RNA is the translational template for ORF2 capsid and the ORF3 viroporin. To test this hypothesis directly, we introduced the same synonymous mutations in the FL Kernow C1 p6 (genotype 3) and FL TW6196E (genotype 4) genomes while retaining the ORF1 protein sequence (Fig. 6a). *In vitro*-transcribed RNAs of the resultant Kernow C1 p6 and TW6196E SgPmut genomes were transfected into Huh7.5 hepatoma cells. Cells were lysed and the lysate supernatant collected at day 5 posttransfection was used to infect Huh7.5 cells, and these were further tested for the presence of infectious particles. RT-quantitative PCR (qPCR) analysis proved that Huh7.5 cells exposed to the SN from FL Kernow C1 p6 wt, but not from the SgPmut (or Pol−) mutant, became infected (Fig. 6b). These data were further corroborated by quantifying HEV ORF2 protein by flow cytometry. Consistent with the HEV RNA quantification, only Huh7.5 cells exposed to the SN from FL Kernow C1 p6 wt, but not from the SgPmut (or Pol−) mutant, expressed ORF2 (Fig. 6c). These data further affirm that positions 5080 to 5123 nt (the numbering is based on M73218 [1a, Burma strain] reference genome) of the HEV genome harbor a region that is critical for subgenomic RNA synthesis and ultimately production and release of infectious virions.

### A genetic element downstream of the transcription start site is also important for subgenomic RNA transcription

**FIG 7:**
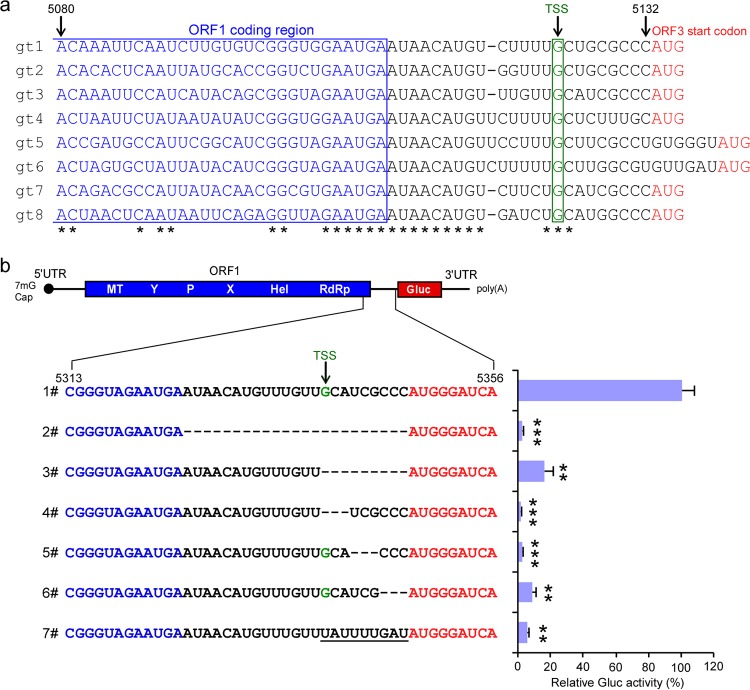
A genetic element downstream of the transcription start site (TSS) is essential for subgenomic RNA transcription. (a) Consensus sequences of the putative promoter (5080 nt to 5132 nt) across genotypes 1 to 8. (b) The truncated mutant viral RNA was transfected into HepG2C3A cells expressing ORF1. Two days posttransfection, supernatants were collected, and *Gaussia* luciferase activity was quantified. The data are presented as the percentage of *Gaussia* luciferase activity relative to that of the full-length rHEVΔORF2/3[Gluc] Pol- (1#). The numbering denotes positions within the viral genome. Values are means plus SD (*n* = 3). *, *P* < 0.05; **, *P* < 0.01; ***, *P* < 0.001 by one-way ANOVA. The numbering of the sequences was referred to M73218 (genotype 1a, Burma strain). The ORF1 coding sequences (blue), the transcriptional start site of HEV subgenomic RNA (green), and the ORF3 start codon (red) are indicated.

Thus far, our data demonstrate that a conserved region from 5080 to 5123 nt of the HEV genome (the numbering is based on M73218 [1a, Burma strain] reference genome) controls synthesis of the SG RNA across multiple different HEV genotypes. Prior seminal work on, e.g., alphaviruses, showed that in addition to a region(s) 5′ of the transcription start site (TSS), residues 3′ of the TSS also serve essential functions for producing Sindbis virus (SINV) subgenomic RNA (18). In the HEV genome, there are 9 nucleotides between the TSS and the translational start side of ORF3 which are highly conserved across all HEV genotypes (Fig. 7a). To test directly whether this region is important for HEV SG RNA synthesis, we generated an rHEVΔORF2/3[Gluc]-Pol- genome lacking the entire +1 through +9 nucleotides. This mutant RNA was subsequently transfected into wt ORF1-expressing HepG2C3A cells, and reporter activity in the supernatant was monitored. Deletion of the +1 to +9 region significantly reduced Gluc activity (Fig. 7b, row 3), suggesting that these additional 9 residues are part of the subgenomic promoter region.

To narrow down further which of the 9 nt are of particular importance, we created three additional rHEVΔORF2/3[Gluc]-Pol- harboring deletions of nt 1 to 3, nt 4 to 6, or nt 7 to 9 and tested them in our transcomplementation assay. Irrespective of the triplet mutation, luminescent activity was greatly reduced (Fig. 7b, rows 4 to 6), indicating that the entire region is important for subgenomic RNA transcription. Collectively, these data demonstrate that both flanking regions of the TSS spanning nucleotides 5295 to 5338 and 5339 to 5347 (+1 through +9) (corresponding to 5080 to 5132 nt of M73218 [1a, Burma strain] reference genome) encompass the promoter for subgenomic RNA synthesis.

## DISCUSSION

**FIG 8:**
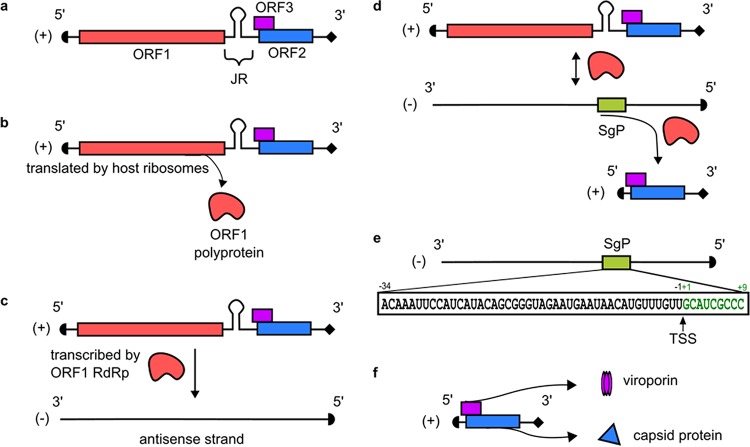
Mechanism of HEV RNA transcription. (a) The HEV genome is a positive-sense (+) single-stranded RNA that is capped at the 5′ end and polyadenylated at the 3′ end. It is organized into three open reading frames (ORFs), where ORF1 contains the RdRp as well as other nonstructural proteins, ORF2 contains the capsid protein, and ORF3 is a viroporin critical for viral release. ORF2 and ORF3 are partially overlapping and are separated from ORF1 by a junctional region (JR) containing a stem-loop structure. (b) Once the viral particle enters the cell and uncoats, ORF1 is translated by host ribosomes into the ORF1 polyprotein containing the RdRp. It is undetermined whether posttranslational processing of the ORF1 polyprotein occurs. (c) The RdRp transcribes a full-length negative-sense (−) intermediate strand. (d) The negative-sense strand is used as a template to produce more full-length (+) RNA to be packaged into progeny virions, as well as a subgenomic (SG) RNA that encodes ORF2 and ORF3. This SG RNA is transcribed from the (−) intermediate strand using a SgP. (e) Sequence for the SgP region based on the Kernow C1/p6 strain of genotype 3 HEV. (f) ORF2 capsid protein and ORF3 viroporin are translated from the SG RNA. These proteins are needed for packaging, assembly, and release of progeny virions.

Here, we shed light on the mechanism by which generation of the subgenomic RNA encoding ORF2 and ORF3 is transcriptionally regulated. Previous data showed that the viral RdRp (possibly acting as part of the ORF1 polyprotein) binds to the 3′ untranslated region (3′ UTR) of HEV and transcribes complementary, full-length (FL) negative-sense (−) viral RNA (19) which serves as a template for transcribing (i) (+) FL HEV RNA and (ii) a 2-kb SG transcript encoding ORFs 2 and 3 (11, 19–21) (Fig. 8). It is still debated whether an additional 3.7-kb SG RNA is transcribed from the (-) sense template (11, 22, 23). Until now, the transcriptional kinetics of the FL and SG RNAs were poorly understood, but it is known that the RdRp has the highest affinity for the 3′ UTR of the (−) sense strand where it begins synthesis of the (+) sense FL RNA, and a lower affinity for the putative SG RNA promoter thought to be confined to the junctional region (JR) between ORFs 1 and 2 (24). The JR contains a highly conserved stem-loop structure, and mutations or deletions in this region completely abolish ORF2 and ORF3 protein synthesis (13, 25). Building on these observations, we mapped precisely the subgenomic promoter governing synthesis of HEV subgenomic RNA using a novel transcomplementation assay for ORF1 function. Notably, the SgP region spans beyond the JR into the 3′ end of the coding region of ORF1 and is further extended to the +9 position following the TSS. This genomic element is highly conserved across all eight HEV genotypes. Taking advantage of a novel panel of HEV reporter genomes of genotypes 1, 3, and 4, we provide direct evidence that mutations within this region affect subgenomic RNA transcription across genetically diverse HEV genomes. Of note, the SgP sequence is not conserved in the 3′ UTR of the (+) FL RNA, suggesting that HEV RdRp-dependent synthesis of the HEV (−) strand is regulated differently. To ascertain that the sequence is functionally relevant for controlling the transcription of viral RNA and not just the expression of Gluc in our reporter genome, we mutated the SgP in the context of the full-length viral genome. Indeed, mutations within the intragenomic SgP abrogated the generation of the HEV SG RNA but did not affect (+) and (−) strand FL RNA synthesis. Since the mutations we introduced were synonymous in nature, the reductions in SG RNA can be directly attributed to this *cis*-acting element on viral RNA transcription, not to any changes in ORF1 function.

In future studies, it will be of great interest to determine the dynamics of the HEV (+) and (−) strand and subgenomic RNAs both longitudinally and across different tissue and cell types. Conceivably, albeit unlikely given the high degree of sequence conservation of the SgP region, HEV genotypes primarily infecting zoonotic hosts (e.g., genotypes 5, 6, 7, and 8) may have different RNA kinetics, which could be tested as we gain access to the appropriate materials and cells from the primary host species (wild boar, camel).

The identification of the subgenomic promoter regions has been facilitated by our new transcomplementation system. By delivering ORF1 function in *trans*, we were able to interrogate the impact of specific nucleotide deletions within the RdRp region of ORF1. Thus, a similar analysis could not have been performed in the context of the FL viral genome. Our system also overcomes several notable challenges to studying HEV, such as its limited replication efficiency *in vitro*. By delivering ORF1 in *trans* with a genome carrying a highly sensitive Gluc reporter, we created a simple readout for evaluating the effect of introducing mutations in the SgP region. We anticipate that this new transcomplementation system will be widely useful to study other aspects of ORF1 function, including but not limited to its effect on host specificity and its ability to interact with different viral elements. ORF1 is not only the largest but arguably also the most complex gene product encoded by HEV. Numerous distinct units have been predicted within ORF1 (4), including an RdRp, RNA helicase, and methyltransferase (26–28) whose functional importance has, however, been only incompletely validated. HEV also contains several other, less well-characterized domains including the “X” and “Y” domains, the hypervariable region (HVR), and a putative papain-like cysteine protease (PCP). HEV plausibly contains a PCP based on a bioinformatic comparison that identified a domain in HEV distantly related to the protease of rubella virus (4). The ability to uncouple HEV RNA transcription from protein translation in our sensitive and easily amenable transcomplementation system will be of great utility to gain additional insights into ORF1 function, and potentially lead to desperately needed therapeutic targets against HEV.

## MATERIALS AND METHODS

### Cell lines and cell culture.

HEK293T cells (American Tissue Culture Collection, ATCC, Manassas, VA, CRL-3216), HepG2C3A cells (ATCC, CRL-10741) and Huh7.5 cells (kindly provided by Charles Rice, The Rockefeller University) were maintained in Dulbecco’s modified Eagle medium (DMEM) (Gibco, NY, USA) supplemented with 10% (vol/vol) fetal bovine serum (FBS), 50 IU/ml penicillin and streptomycin, in a humidified 5% (vol/vol) CO_2_ incubator at 37°C.

### Bioinformatic analysis.

The following hepatitis E virus (HEV) strains were used for sequence alignment: GenBank identifiers [IDs] or accession numbers M73218 (genotype 1a, Burma strain), M74506 (2a, Mex), JQ679013.1 (3, Kernow-C1/p6), AB197673 (4a), AB573435 (5a), AB856243 (6, wbJNN_13), KJ496144 (7, 180C), and KX387866 (8, 48XJ). M73218 (genotype 1a, Burma strain) was used as the reference strain for numbering. A ClustalW multiple-sequence alignment was performed on regions containing the putative subgenomic promoter (SgP), using MacVector software version 15.1.5.

### Plasmid construction.

To construct lentiviral constructs encoding ORF1 of Kernow C1/p6 (GenBank accession number JQ679013), the Kernow C1/p6 ORF1 cDNA was amplified by PCR from a plasmid encoding the full-length (FL) infectious HEV clone Kernow C1/p6 (a kind gift from Suzanne Emerson, NIH) and then cloned into pLVX-IRES-zsGreen1 vector using an In-Fusion HD cloning kit (Clontech, Mountain View, CA, USA). The GAD mutant of ORF1 inactivating the polymerase was generated by QuikChange (Stratagene) site-directed mutagenesis. The pKernow-C1 p6/Gluc (14) was kindly provided by Suzanne Emerson and used to generate the truncation mutants for the mapping of the SgP by QuikChange (Stratagene) site-directed mutagenesis.

### Generation of HEV reporter genomes.

To generate the pSK-SAR55-Gluc reporter construct, PU-O-4337 and PU-O-4338 were used as primers to produce DNA fragment 1 with pSK-SAR55 as the template by PCR, PU-O-2783 and PU-O-3934 were used as primers to produce DNA fragment 2 (containing the Gluc reporter gene) with HEV pKernow-C1 p6-Gluc as the template by PCR, and PU-O-4339 and PU-O-4340 were used as primers to produce DNA fragment 3 with pSK-SAR55 as the template by PCR assay. These three DNA fragments were assembled into pSK-SAR55 (SfiI- and EcoRI-digested) vector. To generate the pSK-SAR55(S17)-Gluc reporter construct, PU-O-4871 and PU-O-4338 were used as primers to produce DNA fragment 1 with pSK-SAR55(S17) as the template by PCR, and PU-O-2783 and PU-O-4340 were used as primers to produce DNA fragment 2 (containing the Gluc reporter gene) with pSK-SAR55-Gluc as the template by PCR. These two DNA fragments were assembled into pSK-SAR55(S17) (EcoRI-digested) vector. To make the pSK-SAR55-Gluc GAD mutant, QuikChange (Stratagene) site-directed mutagenesis was performed using pSK-SAR55-Gluc as the template and PU-O-4635 and PU-O-4636 as the primers.

To generate the pKernow-C1 p6-Gluc reporter construct without the S17 insertion, PU-O-4031 and PU-O-4261 were used as the primers to produce DNA fragment 1 using pKernow-C1 p6-Gluc as the template by PCR, and PU-O-4260 and PU-O-4322 were used as the primers to produce DNA fragment 2 using pKernow-C1 p6-Gluc as the template by PCR assay. Both DNA fragments were assembled into pKernow-C1 p6-Gluc vector (XbaI, BamHI, and AflII digested). To make the pKernow-C1 p6-Gluc GAD construct, QuikChange (Stratagene) site-directed mutagenesis was performed using pKernow-C1 p6-Gluc as the template and PU-O-2711 and PU-O-2712 as the primers.

To generate the pGEM-9Zf-pSHEV3-Gluc reporter construct, PU-O-4327 and PU-O-4328 were used as primers to produce DNA fragment 1 using pGEM-9Zf-pSHEV3 as the template by PCR assay, and PU-O-2783 and PU-O-4329 were used as the primers to produce DNA fragment 2 (containing the Gluc gene) using Kernow-C1 p6/Gluc as the template by PCR assay. These DNA fragments assembled into pGEM-9Zf-pSHEV3 (AflII- and PmlI-digested) vector. To generate the pGEM-9Zf-pSHEV3(S17)-Gluc reporter construct, PU-O-4844 and PU-O-4845 were used as primers to produce DNA fragment 1 with pGEM-9Zf-pSHEV3-Gluc as the template by PCR, PU-O-4846 and PU-O-4847 were used as primers to produce DNA fragment 2 (containing the Gluc reporter gene) with HEV pKernow-C1 p6/Gluc as the template by PCR, and PU-O-4848 and PU-O-4849 were used as primers to produce DNA fragment 3 with pGEM-9Zf-pSHEV3-Gluc as the template by PCR assay. These three DNA fragments were assembled into pGEM-9Zf-pSHEV3-Gluc (StuI-digested) vector. To make the pGEM-9Zf-pSHEV3-Gluc GAD construct, QuikChange (Stratagene) site-directed mutagenesis was performed using pGEM-9Zf-pSHEV3-Gluc as the template and PU-O-4376 and PU-O-4377 as the primers.

To generate the pGEM-7Zf(-)-TW6196E-Gluc reporter construct, PU-O-4330 and PU-O-4331 were used as primers to produce DNA fragment 1 using pGEM-7Zf(-)-TW6196E as the template by PCR assay, PU-O-2783 and PU-O-3934 were used as the primers to produce DNA fragment 2 (containing the Gluc gene) using Kernow-C1 p6/Gluc as the template by PCR assay, and PU-O-4332 and PU-O-4333 were uses as the primers to produce DNA fragment 3 using pGEM-7Zf(-)-TW6196E as the template by PCR assay. These DNA fragments assembled into the pGEM-7Zf(-)-TW6196E (Sbf-I and EcoRI-digested) vector. To generate the pGEM-7Zf(-)-TW6196E(S17)-Gluc reporter construct, PU-O-4850 and PU-O-4851 were used as primers to produce DNA fragment 1 using pGEM-7Zf(-)-TW6196E-Gluc as the template by PCR assay, and DNA fragment 2 containing S17 element and partial pGEM-7Zf(-)-TW6196E sequences were synthesized. These DNA fragments were assembled in the pGEM-7Zf(-)-TW6196E-Gluc (ApaI digested) vector. To generate the pGEM-7Zf(-)-TW6196E-Gluc GAD construct, QuikChange (Stratagene) site-directed mutagenesis was performed using pGEM-7Zf(-)-TW6196E-Gluc as the template and PU-O-4631 and PU-O-4632 as the primers.

All DNA fragments were cloned into the respective vectors using an In-Fusion HD cloning kit (Clontech, Mountain View, CA, USA). All constructs or primers used to construct the HEV reporter genome in Table 1 have been validated through Sanger sequencing and are available upon request.

**TABLE 1:** Primers used to construct the HEV reporter genome

Primer	Sequence (5′–3′)
PU-O-2711	CTGCCTTTAAGGGTGCTGATTCGGTGGTCCT
PU-O-2712	AGGACCACCGAATCAGCACCCTTAAAGGCAG
PU-O-2783	ATGGGAGTCAAAGTTCTGTTTGC
PU-O-3934	GTCACCACCGGCCCCCTTGATCTTG
PU-O-4031	CACCGCGGTGGCTAGCGCTCTAGATAATACGACTCACT
PU-O-4260	CGCCCTCCGAGGAGTCTCAGGTCGATGCAG
PU-O-4261	CTGCATCGACCTGAGACTCCTCGGAGGGCG
PU-O-4322	GAATGCTTCTTCCAGAAACCCTTAAG
PU-O-4327	CAGGCGCCGAAGGAGTCTCTTAAGGGTTTC
PU-O-4328	AACAGAACTTTGACTCCCATGGTGATCCCATGGGCGATGC
PU-O-4329	TGCCGGCGCAGGATAGCACCACGTGAG
PU-O-4330	CTCCGCCTGGGTCCTGCAGG
PU-O-4331	AACAGAACTTTGACTCCCATGGTGGCATCGCCATGCAA
PU-O-4332	TCAAGGGGGCCGGTGGTGACTAATTCGCGTGGTGCTATACTGC
PU-O-4333	GGACATCGGTGGAAGTGATGGAATTC
PU-O-4337	TGCTGTCCACGCTCGTGGGCCGTTATGGCC
PU-O-4338	AACAGAACTTTGACTCCCATGGTCGCGAACCCATGGGCGC
PU-O-4339	TCAAGGGGGCCGGTGGTGACTAACTCCCGCGGCGCCATCCT
PU-O-4340	GGACATCCGTCGAGGTTATTGAATTC
PU-O-4376	CCGCCTTCAAGGGTGCTGATTCGGTGGTCCT
PU-O-4377	AGGACCACCGAATCAGCACCCTTGAAGGCGG
PU-O-4631	CGGCATTTAAAGGGGcTGACTCTGTTGTGCT
PU-O-4632	AGCACAACAGAGTCAGCCCCTTTAAATGCCG
PU-O-4635	CTGCCTTTAAAGGTGCTGATTCGATAGTGCT
PU-O-4636	AGCACTATCGAATCAGCACCTTTAAAGGCAG
PU-O-4844	CTATGGCCATGACAACGAGGCCTATGAGGG
PU-O-4845	GTTGCCCAGGCGCGTGTAGCACTCCTCTGAAGGTGG
PU-O-4846	GCTACACGCGCCTGGGCAACG
PU-O-4847	CCTCCTCCTGCAGCTTGATG
PU-O-4848	CATCAAGCTGCAGGAGGAGGCTCAGGTCGATGCGGCAT
PU-O-4849	CTGGCAGGACGGCGGGAAGGCCTG
PU-O-4850	GTCAGTGAATGGGGCGAATTGGGCCCGACGTCG
PU-O-4851	GTTGCCCAGGCGCGTGTAGCGCTGAGTCTGCACAGGGG
PU-O-4871	TGTCACGCATCTGATGAAGCGAATTCAG

### Lentivirus production.

Vesicular stomatitis virus G protein (VSV-G) pseudotyped lentiviruses were produced by transient cotransfection of the third-generation packaging plasmids pMD2G (catalog no. 12259; Addgene) and psPAX2 (catalog number 12260; Addgene) and the transfer vector with XtremeGENE HP DNA transfection reagent (Sigma-Aldrich, St. Louis, MO, USA) into HEK293T cells. The medium was changed 6 h posttransfection. Supernatants were collected at 48 and 72 h after transfection, pooled, passed through a 0.45-µm filter, aliquoted, and frozen at −80°C. For lentiviral transduction, HepG2C3A cells (1 × 10^5^ cells/well) were seeded in collagen-coated six-well tissue culture plates and infected the following day with lentiviruses. To determine the transduction efficiency, cells were trypsinized and processed for flow cytometric analysis 3 days following transduction.

### Flow cytometry analysis.

Expression of lentivirally delivered ORF1 or ORF1 GAD was analyzed by flow cytometry on a BD LSRII flow cytometer (five-laser SORP LSRII with high-throughput sampler). Kernow C1/p6 ORF1 or ORF1 GAD were transduced into target cells by bicistronic lentiviruses expressing zsGreen. After 3 days of transduction, cells were fixed in 4% (wt/vol) paraformaldehyde (PFA) in phosphate-buffered saline (PBS) for 15 min, following by two washes with PBS. The efficiencies of transduction of transgenes were determined by simultaneous expression of zsGreen. Quantification of the HEV-infected cells was performed using ORF2-specific convalescent-phase serum collected from an HEV-infected chimpanzee (CH1313, kindly provided by Suzanne Emerson [National Institutes of Health, Bethesda, MD]). Briefly, supernatant from the HEV RNA-transfected cells was used to inoculate naive Huh 7.5 cells for 12 h. The cells were washed with PBS three times and subsequently cultured in DMEM with 10% (vol/vol) FBS. After 3 days of infection, the cells were trypsinized, and single-cell suspensions were fixed with 4% (wt/vol) PFA and permeabilized in PBS plus 0.01% Triton X-100, following two washes with PBS. The cells were stained with anti-ORF2 serum CH1313 (1:100) for 1 h at room temperature, followed by two washes with PBS. Cells were subsequently incubated with goat anti-human IgG secondary antibody conjugated with Alexa Fluor 488 (Life Technologies) at a 1:1,000 dilution for 1 h at room temperature. All samples were analyzed on a BD LSRII flow cytometer using FlowJo software.

### *In vitro* transcription assay and viral RNA transfection.

HEV Kernow-C1 p6, HEV Kernow-C1 p6-Gluc, and the truncated mutant plasmids were linearized by MluI. pSAR55-Gluc was linearized by BglII, pGEM-9Zf-pSHEV3-Gluc was linearized by XbaI, and pGEM-7Zf(-)-TW6196E and pGEM-7Zf(-)-TW6196E-Gluc were linearized by SpeI. Viral capped RNAs were transcribed *in vitro* from linearized plasmid using HiScribe T7 antireverse cap analog (ARCA) mRNA kit (New England Biolabs, Ipswich, MA) according to the manufacturer’s instructions. The *in vitro* transcription (IVT) reaction mixture of 20 µl was assembled by adding DNA template (1 µg), 10 µl of 2×ARCA/nucleotide triphosphate (NTP) mix and 2 µl of T7 RNA polymerase Mix. The reaction mixture was incubated at 37°C for 2 h, and 1 µl of DNase was added to the IVT reaction mixture and incubated for 15 min at 37°C to remove the template DNA. Then, the viral RNA was purified by LiCl precipitation. Viral RNA was transfected into HepG2C3A cells or Huh7.5 cells using TransIT-mRNA transfection reagent (Mirus Bio LLC, Madison, WI) according to the instructions.

### *Gaussia* luciferase assays.

*Gaussia* luciferase activity was determined using Luc-Pair Renilla luciferase HS assay kit (GeneCopoeia, Rockville, MD). Specifically, 10 µl of harvested cell culture medium was added per well of a 96-well solid white, flat-bottom polystyrene microplate (Corning, NY, USA), followed by the addition of Renilla luciferase assay substrate and the detection of luminescence was performed using a Berthold luminometer.

### HEV production and infection.

The *in vitro*-transcribed viral capped RNAs (2 µg) were transfected into Huh7.5 cells (2 × 10^5^ cells) in the six-well tissue culture plates using TransIT-mRNA transfection reagent (Mirus Bio LLC, Madison, WI). Medium was removed from transfected cells after 5 days transfection, and cells were trypsinized and washed with PBS once. Cell pellets were extracted by adding 1 ml double-distilled (DDI) water per well and vortexing vigorously until the cell pellets were completely suspended. The sample was put on ice for 30 min, vortexing intermittently every 10 min. The samples were centrifuged at 13,000 × *g* for 10 min to remove cellular debris. Supernatant (0.9 ml) was transferred to a new tube, and 0.1 ml of 10× PBS was added. The supernatant was stocked at −80°C. As for the infection assay, the Huh7.5 cells were seeded into a 24-well tissue culture plate 24 h prior to infection, and cells were infected by HEV the following day, and medium was changed 12 h after the infection. After 3 days, cells were collected for further analysis.

### Preparation of RNA standard for reverse transcription-quantitative PCR (RT-qPCR) assay.

To prepare the RNA standard used to quantify the Kernow C1/p6 Gluc negative-strand RNA abundance, DNA fragment spanning from 4751 nt to 6924 nt of Kernow C1/p6 Gluc was amplified by PCR and then subcloned into the pCR4BluntTOPO vector (Thermo Fisher Scientific) under the control of the T7 promoter. The correct insert direction clones were identified by DNA sequencing as the templates for an *in vitro* transcription assay to produce the negative-strand viral RNA. The *in vitro* transcription assay was performed using HiScribe T7 ARCA mRNA kit (New England Biolabs) as described above. The RNA templates were purified and stocked at −80°C.

### Quantification of HEV (−) strand RNA by RT-qPCR assay.

Series-diluted predetermined copies of standard RNA or 100 ng of total RNA from each sample were used for reverse transcription using SuperScript III reverse transcriptase (Invitrogen, NY, USA). The reverse transcription primer (PU-O-4508) contained a nonviral tag sequence attached at the 5′ end of the strand-specific viral sequence that was used to prime reverse transcription (Table 2). The RT reaction was assembled with 5× first-strand buffer, 20 mM dithiothreitol (DTT), 0.5 mM deoxynucleoside triphosphates (dNTPs), 100 nM RT primer, 1 µl RNaseOUT recombinant RNase inhibitor, and 2 U of SuperScript III reverse transcriptase in a volume of 20 µl. The reaction was carried out at 55°C for 30 min and inactivated by heating at 70°C for 15 min and then diluted to 100 µl (1:1) with nuclease-free water for real-time qPCR.

**TABLE 2:** Primers used for RT-qPCR to quantify Kernow C1 p6 Gluc negative-strand RNA

Primer	Sequence (5′–3′)^a^
PU-O-4508	CGGGAAGGCGACTGGAGTGCCTCTGTGTGTGGACTGCACAA
PU-O-4509	TGGATCTTGCTGGCAAAGGT
PU-O-4125	CGGGAAGGCGACTGGAGTGCC

a The unique nonviral tag sequence in the reverse transcription primer (PU-O-4508) sequence is underlined.

Real-time qPCRs was prepared using SYBR green PCR master mix (Applied Biosystems, CA, USA). Briefly, 2 µl of diluted cDNA was mixed with 2×SYBR green PCR master mix and the primers (PU-O-4509 and PU-O-4125) (Table 2) at a final concentration of 125 nM prior to enzyme activation by incubation at 95°C for 10 min. The reaction mixtures were then subjected to 40 cycles of PCR, with 1 cycle consisting of 15 s at 95°C and 1 min at 60°C. The standard curve was generated using *in vitro*-transcribed standard RNA. The viral genome copy numbers were determined by interpolation of the standard curve for the respective strand of RNA.

### Statistical analysis.

Student’s *t* test or one-way analysis of variance (ANOVA) with Tukey’s honestly significant difference (HSD) test was used to test for statistical significance of the differences between the different group parameters. *P* values of less than 0.05 were considered statistically significant.

## References

[1] Balayan MS, Andjaparidze AG, Savinskaya SS, Ketiladze ES, Braginsky DM, Savinov AP, Poleschuk VF 1983 Evidence for a virus in non-A, non-B hepatitis transmitted via the fecal-oral route. Intervirology 20:23–31. doi:10.1159/000149370. 6409836

[2] Nimgaonkar I, Ding Q, Schwartz RE, Ploss A 2018 Hepatitis E virus: advances and challenges. Nat Rev Gastroenterol Hepatol 15:96–110. doi:10.1038/nrgastro.2017.150. 29162935 PMC11329273

[3] Reyes GR, Purdy MA, Kim JP, Luk KC, Young LM, Fry KE, Bradley DW 1990 Isolation of a cDNA from the virus responsible for enterically transmitted non-A, non-B hepatitis. Science 247:1335–1339. doi:10.1126/science.2107574. 2107574

[4] Koonin EV, Gorbalenya AE, Purdy MA, Rozanov MN, Reyes GR, Bradley DW 1992 Computer-assisted assignment of functional domains in the nonstructural polyprotein of hepatitis E virus: delineation of an additional group of positive-strand RNA plant and animal viruses. Proc Natl Acad Sci U S A 89:8259–8263. doi:10.1073/pnas.89.17.8259. 1518855 PMC49897

[5] Yin X, Ambardekar C, Lu Y, Feng Z 2016 Distinct entry mechanisms for nonenveloped and quasi-enveloped hepatitis E viruses. J Virol 90:4232–4242. doi:10.1128/JVI.02804-15. 26865708 PMC4810531

[6] Nair VP, Anang S, Subramani C, Madhvi A, Bakshi K, Srivastava A, Shalimar, Nayak B, Ranjith Kumar CT, Surjit M 2016 Endoplasmic reticulum stress induced synthesis of a novel viral factor mediates efficient replication of genotype-1 hepatitis E virus. PLoS Pathog 12:e1005521. doi:10.1371/journal.ppat.1005521. 27035822 PMC4817972

[7] Neuvonen M, Ahola T 2009 Differential activities of cellular and viral macro domain proteins in binding of ADP-ribose metabolites. J Mol Biol 385:212–225. doi:10.1016/j.jmb.2008.10.045. 18983849 PMC7094737

[8] Anang S, Subramani C, Nair VP, Kaul S, Kaushik N, Sharma C, Tiwari A, Ranjith-Kumar CT, Surjit M 2016 Identification of critical residues in hepatitis E virus macro domain involved in its interaction with viral methyltransferase and ORF3 proteins. Sci Rep 6:25133. doi:10.1038/srep25133. 27113483 PMC4844956

[9] Ojha NK, Lole KS 2016 Hepatitis E virus ORF1 encoded macro domain protein interacts with light chain subunit of human ferritin and inhibits its secretion. Mol Cell Biochem 417:75–85. doi:10.1007/s11010-016-2715-0. 27170377 PMC7089094

[10] Ding Q, Heller B, Capuccino JM, Song B, Nimgaonkar I, Hrebikova G, Contreras JE, Ploss A 2017 Hepatitis E virus ORF3 is a functional ion channel required for release of infectious particles. Proc Natl Acad Sci U S A 114:1147–1152. doi:10.1073/pnas.1614955114. 28096411 PMC5293053

[11] Graff J, Torian U, Nguyen H, Emerson SU 2006 A bicistronic subgenomic mRNA encodes both the ORF2 and ORF3 proteins of hepatitis E virus. J Virol 80:5919–5926. doi:10.1128/JVI.00046-06. 16731930 PMC1472559

[12] Ichiyama K, Yamada K, Tanaka T, Nagashima S, Jirintai, Takahashi M, Okamoto H 2009 Determination of the 5′-terminal sequence of subgenomic RNA of hepatitis E virus strains in cultured cells. Arch Virol 154:1945–1951. doi:10.1007/s00705-009-0538-y. 19885718

[13] Cao D, Huang YW, Meng XJ 2010 The nucleotides on the stem-loop RNA structure in the junction region of the hepatitis E virus genome are critical for virus replication. J Virol 84:13040–13044. doi:10.1128/JVI.01475-10. 20943962 PMC3004356

[14] Shukla P, Nguyen HT, Torian U, Engle RE, Faulk K, Dalton HR, Bendall RP, Keane FE, Purcell RH, Emerson SU 2011 Cross-species infections of cultured cells by hepatitis E virus and discovery of an infectious virus-host recombinant. Proc Natl Acad Sci U S A 108:2438–2443. doi:10.1073/pnas.1018878108. 21262830 PMC3038723

[15] Emerson SU, Zhang M, Meng XJ, Nguyen H, St Claire M, Govindarajan S, Huang YK, Purcell RH 2001 Recombinant hepatitis E virus genomes infectious for primates: importance of capping and discovery of a cis-reactive element. Proc Natl Acad Sci U S A 98:15270–15275. doi:10.1073/pnas.251555098. 11742081 PMC65019

[16] Huang YW, Haqshenas G, Kasorndorkbua C, Halbur PG, Emerson SU, Meng XJ 2005 Capped RNA transcripts of full-length cDNA clones of swine hepatitis E virus are replication competent when transfected into Huh7 cells and infectious when intrahepatically inoculated into pigs. J Virol 79:1552–1558. doi:10.1128/JVI.79.3.1552-1558.2005. 15650181 PMC544089

[17] Córdoba L, Feagins AR, Opriessnig T, Cossaboom CM, Dryman BA, Huang YW, Meng XJ 2012 Rescue of a genotype 4 human hepatitis E virus from cloned cDNA and characterization of intergenotypic chimeric viruses in cultured human liver cells and in pigs. J Gen Virol 93:2183–2194. doi:10.1099/vir.0.043711-0. 22837416 PMC3541786

[18] Wielgosz MM, Raju R, Huang HV 2001 Sequence requirements for Sindbis virus subgenomic mRNA promoter function in cultured cells. J Virol 75:3509–3519. doi:10.1128/JVI.75.8.3509-3519.2001. 11264340 PMC114842

[19] Agrawal S, Gupta D, Panda SK 2001 The 3′ end of hepatitis E virus (HEV) genome binds specifically to the viral RNA-dependent RNA polymerase (RdRp). Virology 282:87–101. doi:10.1006/viro.2000.0819. 11259193

[20] Nanda SK, Panda SK, Durgapal H, Jameel S 1994 Detection of the negative strand of hepatitis E virus RNA in the livers of experimentally infected rhesus monkeys: evidence for viral replication. J Med Virol 42:237–240. doi:10.1002/jmv.1890420306. 7516419

[21] Varma SP, Kumar A, Kapur N, Durgapal H, Acharya SK, Panda SK 2011 Hepatitis E virus replication involves alternating negative- and positive-sense RNA synthesis. J Gen Virol 92:572–581. doi:10.1099/vir.0.027714-0. 21123540

[22] Xia X, Huang R, Li D 2000 Studies on the subgenomic RNAs of hepatitis E virus. Wei Sheng Wu Xue Bao 40:622–627. (In Chinese.). 12549057

[23] Yarbough PO, Tam AW, Fry KE, Krawczynski K, McCaustland KA, Bradley DW, Reyes GR 1991 Hepatitis E virus: identification of type-common epitopes. J Virol 65:5790–5797. 1717709 10.1128/jvi.65.11.5790-5797.1991 PMC250240

[24] Mahilkar S, Paingankar MS, Lole KS 2016 Hepatitis E virus RNA-dependent RNA polymerase: RNA template specificities, recruitment and synthesis. J Gen Virol 97:2231–2242. doi:10.1099/jgv.0.000528. 27324050

[25] Huang YW, Opriessnig T, Halbur PG, Meng XJ 2007 Initiation at the third in-frame AUG codon of open reading frame 3 of the hepatitis E virus is essential for viral infectivity in vivo. J Virol 81:3018–3026. doi:10.1128/JVI.02259-06. 17202216 PMC1866010

[26] Fry KE, Tam AW, Smith MM, Kim JP, Luk KC, Young LM, Piatak M, Feldman RA, Yun KY, Purdy MA, McCaustland KA, Bradley DW, Reyes GR 1992 Hepatitis E virus (HEV): strain variation in the nonstructural gene region encoding consensus motifs for an RNA-dependent RNA polymerase and an ATP/GTP binding site. Virus Genes 6:173–185. doi:10.1007/BF01703066. 1589964

[27] Rozanov MN, Koonin EV, Gorbalenya AE 1992 Conservation of the putative methyltransferase domain: a hallmark of the ‘Sindbis-like’ supergroup of positive-strand RNA viruses. J Gen Virol 73:2129–2134. doi:10.1099/0022-1317-73-8-2129. 1645151

[28] Magden J, Takeda N, Li T, Auvinen P, Ahola T, Miyamura T, Merits A, Kääriäinen L 2001 Virus-specific mRNA capping enzyme encoded by hepatitis E virus. J Virol 75:6249–6255. doi:10.1128/JVI.75.14.6249-6255.2001. 11413290 PMC114346

