# Supplementary figures and images for: Identification of the Intragenomic Promoter Controlling Hepatitis E Virus Subgenomic RNA Transcription
Source: mBio. 2018 May 8;9(3):e00769-18. doi: 10.1128/mBio.00769-18 (PMC5941075; doi:10.1128/mBio.00769-18)

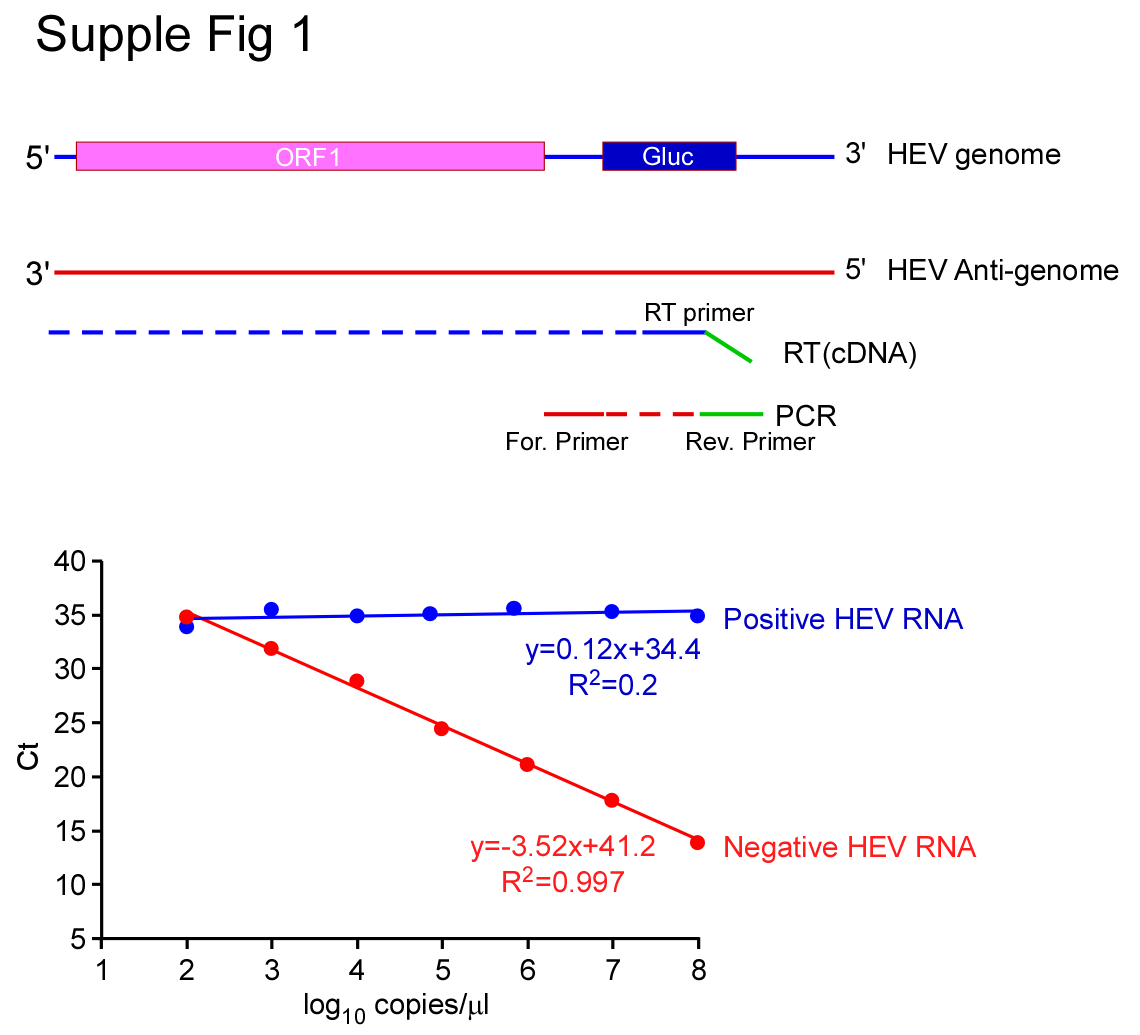

Supplement: FIG S1 [file mbo003183872sf1.tif]

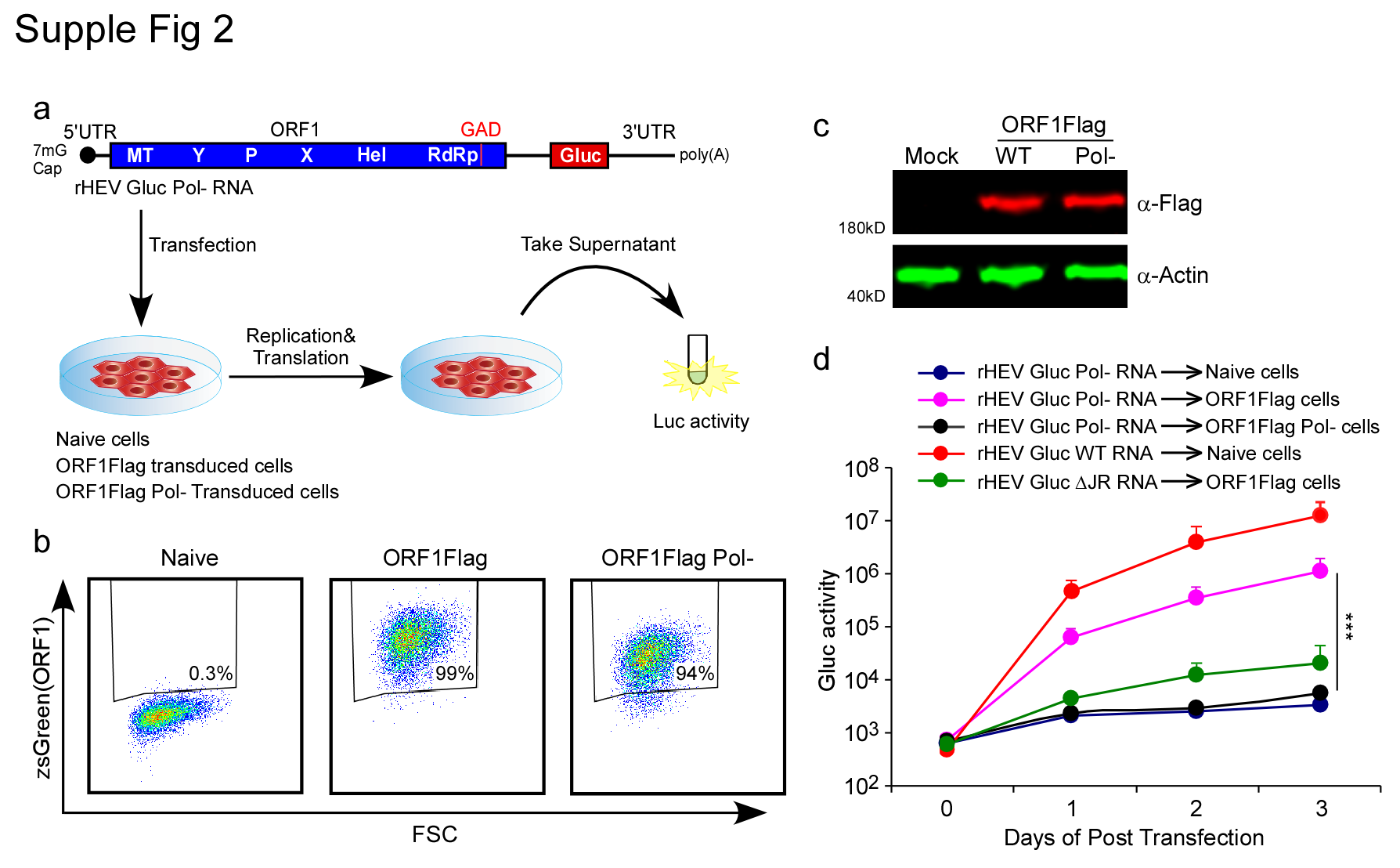

Supplement: FIG S2 [file mbo003183872sf2.tif]

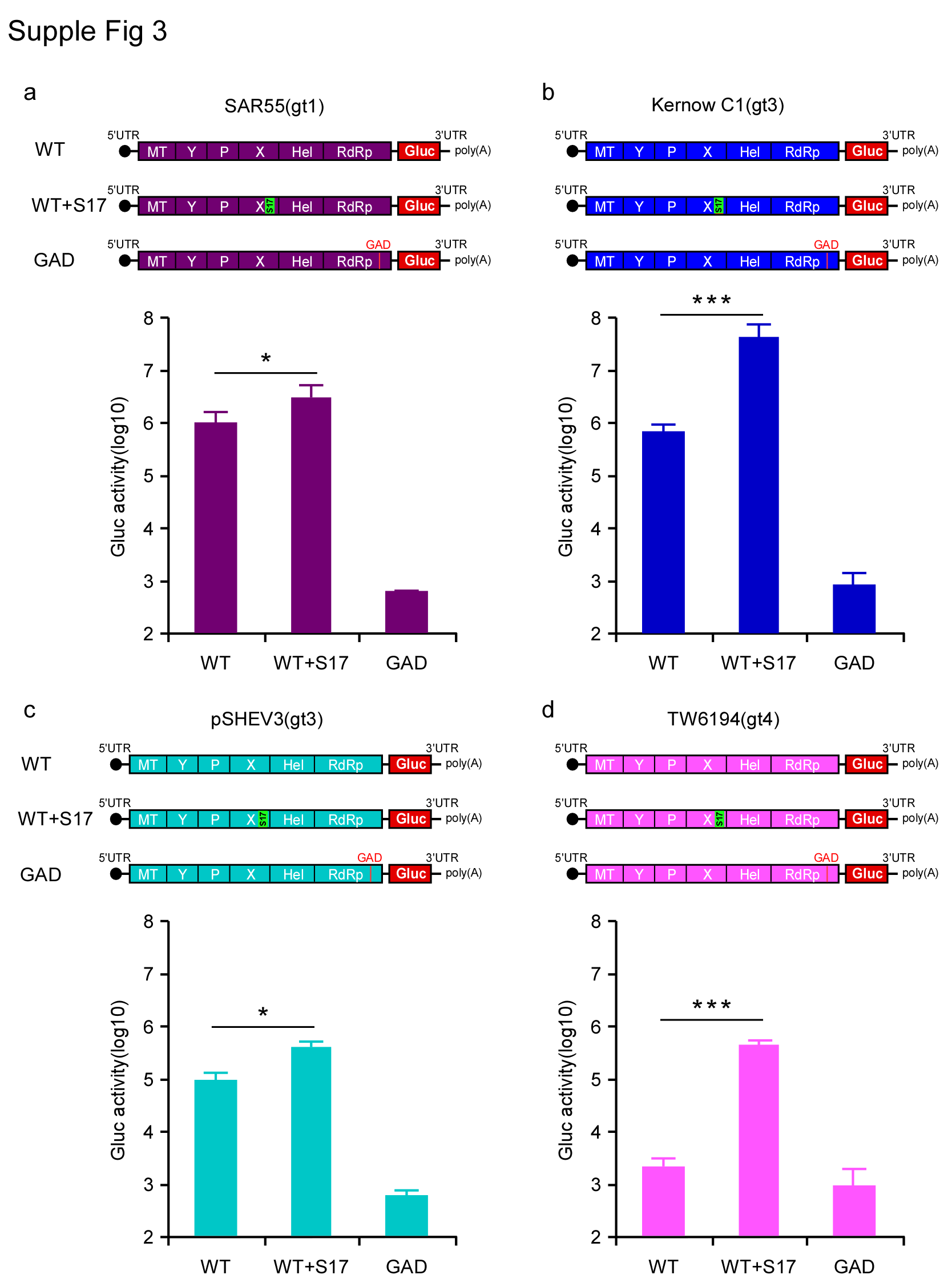

Supplement: FIG S3 [file mbo003183872sf3.tif]
